# Correlation of skin rash and overall survival in patients with pancreatic cancer treated with gemcitabine and erlotinib – results from a non-interventional multi-center study

**DOI:** 10.1186/s12885-020-6636-7

**Published:** 2020-02-24

**Authors:** C. Benedikt Westphalen, Tobias Kukiolka, Benjamin Garlipp, Lars Hahn, Martin Fuchs, Peter Malfertheiner, Marcel Reiser, Fabian Kütting, Volker Heinemann, Andreas Beringer, Dirk T. Waldschmidt

**Affiliations:** 1 Comprehensive Cancer Center Munich & Department of Medicine III, University Hospital, LMU Munich, Marchioninistr. 15, 81377 Munich, Germany; 2 Department of Medicine I, University Hospital, Ulmenweg 18, 91054 Erlangen, Germany; 3 Department of Surgery, Otto-von-Guericke University Magdeburg, Magdeburg, Germany; 4 DOKUSAN GmbH & CO. KG, Herne, Germany; 5 Munich Municipal Hospital Group GmbH, Englschalkinger Str. 77, 81925 Munich, Germany; 6 University Hospital Magdeburg, Leipziger Str. 44, 39120 Magdeburg, Germany; 7 PIOH Praxis Internistischer Onkologie und Hämatologie, Richard-Wagner-Str. 13-17, 50674 Cologne, Germany; 8 Department of Gastroenterology and Hepatology, University of Cologne, Kerpener Str. 62, 50937 Cologne, Germany; 9 Roche Pharma AG, Emil-Barell-Str. 1, Grenzach-Wyhlen, Germany

**Keywords:** Pancreatic ductal adenocarcinoma, Gemcitabine, Erlotinib, Overall survival, Progression-free survival, Skin rash, Non-interventional study

## Abstract

**Background:**

Gemcitabine/erlotinib treatment offers limited benefit in unselected patients with pancreatic ductal adenocarcinoma (PDAC). Development of skin rash has been associated with favorable outcomes in patients treated with gemcitabine/erlotinib. This study aimed to extend knowledge on the effectiveness of gemcitabine/erlotinib in metastatic PDAC in the context of clinical practice and with focus on skin rash.

**Methods:**

This multicenter, non-interventional study enrolled 376 patients with metastatic PDAC receiving gemcitabine/erlotinib. The primary endpoint was overall survival (OS) in patients with skin rash versus no skin rash. Secondary endpoints included progression-free survival (PFS), treatment satisfaction and safety. All data were analyzed using descriptive statistics. Survival time and time to disease progression were estimated using the Kaplan-Meier method. Effectiveness endpoints were analyzed for subgroups by skin rash grade (no rash, rash grade 1, rash grade ≥ 2), duration of erlotinib treatment (≤8 weeks, > 8 weeks), Eastern Cooperative Oncology Group (ECOG) performance status at baseline (0–1, 2) and age (≤65 years, > 65 years).

**Results:**

Within the full analysis set (FAS; *N* = 270), 48 patients (17.8%) developed grade 1 rash, 51 patients (18.9%) grade ≥ 2 rash, while 171 patients (63.3%) did not develop a rash. Median OS of all patients was 9.11 months with an OS of 9.93 months in rash-positive and 8.68 months in rash-negative patients. Median PFS was 5.06 months for rash-positive and 4.11 months for rash-negative patients. PFS was longer in patients with rash grade ≥ 2 and in older patients (> 65 years). Examination using a multivariate Cox proportional model revealed that an age > 65 years was associated with longer OS (hazard ratio 0.640; *p* = 0.0327) and PFS (hazard ratio 0.642; *p* = 0.0026). Out of the 338 patients in the SAF, 310 patients (91.7%) experienced at least one AE, and 176 patients (52.1%) experienced skin-related side effects, all of which were CTC grade 1 to 3.

**Conclusions:**

Comparing rash-positive with rash-negative patients showed no significant difference in survival. While patients with rash grade ≥ 2 and older patients (independent of skin reactions) showed longer PFS, this did not translate into prolonged OS. The study did not reveal new safety signals.

**Trial registration:**

ClinicalTrials.gov Identifier: NCT01782690, retrospectively registered on 4 February 2013.

## Background

Pancreatic ductal adenocarcinoma (PDAC) remains an almost uniformly lethal disease with the highest case fatality rate of all major cancers. Five-year overall survival remains in the single digits and has not changed considerably in the last four decades [1, 2]. Alarmingly, pancreatic cancer is on the rise in the United States and Germany and will become the second most common cause of cancer-related death in 2030 [3, 4]. Based on a landmark study in 1997, monotherapy with gemcitabine has been the treatment of choice in patients diagnosed with pancreatic cancer for decades [5].

Consequently, multiple gemcitabine-based combinations have been tested in clinical trials but showed little or no additional benefit [6]. In 2007, the combination of gemcitabine/erlotinib provided a statistically significant but clinically marginal effect on overall survival in patients diagnosed with metastatic pancreatic cancer when compared to gemcitabine plus placebo (6.24 vs. 5.91 months, hazard ratio = 0.82, *p* = 0.038). Based on this study, erlotinib received marketing authorization for the treatment of pancreatic cancer [7]. While the activity of erlotinib is modest in unselected pancreatic cancer patients, individuals developing cutaneous reactions under treatment with erlotinib display favorable clinical outcomes [7–11].

Only recently, the FOLFIRINOX regime and the combination of gemcitabine/nab-paclitaxel proved superior to single agent gemcitabine and are thus considered standard first-line treatment options in metastatic pancreatic cancer [12, 13]. These more efficient cytotoxic regimens come at the cost of higher toxicities [14]. Consequently, with limited treatment options at hand, it is still warranted to assess the effectiveness of the well tolerated combination of gemcitabine/erlotinib in correlation with emerging skin rash. Accordingly, the study presented here aimed to evaluate the clinical effectiveness based on cutaneous reactions and the safety of gemcitabine/erlotinib in a non-interventional, multicenter, phase IV setting.

## Methods

This was a multicenter, non-interventional study in accordance with §4 (23) of the German Drug Law (Arzneimittelgesetz, AMG). The study was approved by the ethical committee of the Faculty of Medicine of the University in Cologne, Germany, as well as by ethical committees in all participating German centers and was conducted according to the Declaration of Helsinki, the Good Clinical Practice guidelines of the International Conference on Harmonization (now: International Council for Harmonization), and relevant German and European laws. Patients with metastatic pancreatic carcinoma were treated with gemcitabine/erlotinib (Tarceva®) based on the decision of the treating physician. Data were collected before the initiation of the treatment (baseline), during treatment and at the end of the observational period (final documentation/end-of-treatment). The maximum duration of documentation for any individual patient was 12 months.

Based on previous studies, it was assumed that the proportion of patients with and without rash would be 2:1. To show a 2.5 months difference in overall survival between patients with and without rash (at a power of 80% and a two-sided significance level of 5%) a sample size of 309 patients was planned. This sample size was deemed sufficient to demonstrate a difference of 18% in the disease control rate (DCR) (estimated DCR of 40% in patients without rash). Survival time and the time to progression of disease were estimated using the Kaplan-Meier method. For the median time to onset of the events, 95% confidence intervals were calculated. The effect of potential influencing and confounding factors on target variables was estimated using a Cox proportional hazard model.

The primary endpoint of this study was overall survival stratified by rash. Median survival was calculated as survival from the date of the first dose of erlotinib. Secondary endpoints included response rates, time to progression, self-reported treatment satisfaction, clinical effectiveness in patients with ECOG performance status 2 and various safety variables. Adverse events (AEs) and serious adverse events (SAEs) were graded and recorded according to the *Common Terminology Criteria for Adverse Events* (CTCAE, v4.0).

## Results

### Study population

**Fig. 1:**
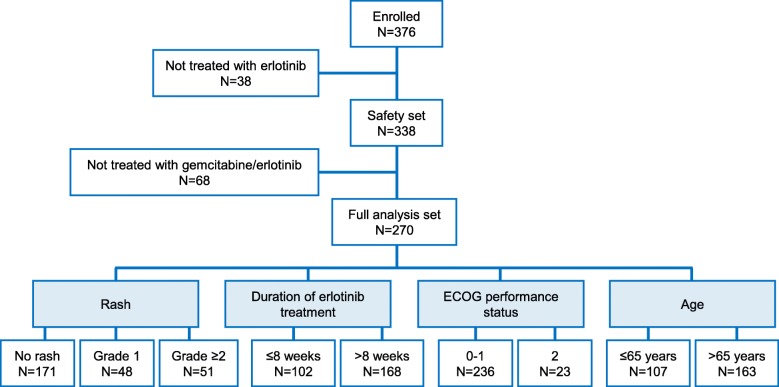
Patient disposition

Between 2012 and 2015, 376 patients were enrolled across 85 German study sites. Out of these, 338 patients received at least one dose of erlotinib and constituted the safety analysis set (SAF). 270 patients commenced treatment with gemcitabine/erlotinib (according to therapy guidance) and comprised the full analysis set (FAS). The median duration from diagnosis of metastatic disease until informed consent was 27 days. Fifty-eight patients (21.5%) in the FAS had received previous palliative chemotherapy before treatment with gemcitabine/erlotinib potentially explaining the mean duration of 71 days from occurrence of metastatic disease until informed consent for this non-interventional study. The study flow is depicted in Fig. 1. Baseline demographics and disease characteristics are shown in Table 1 and Table 2.

**Table 1:** Patient characteristics at baseline

		FAS (*N* = 270)	SAF (*N* = 338)
Age [years]	n (number of patients)	270	338
	Mean (SD)	66.9 (9.4)	66.9 (9.1)
	Median (Min, Max)	69 (38, 83)	69 (38, 83)
Body mass index [kg/m^2^]	n (number of patients)	270	338
	Mean (SD)	24.2 (4.2)	24.4 (4.3)
	Median (Min, Max)	24 (15, 47)	24 (15, 47)
Sex [% (n)] ^a^	Male	59.3 (160)	60.1 (203)
	Female	40.7 (110)	39.9 (135)
Smoking status [n (%)] ^a^	Non-smoker	177 (65.6)	230 (68.0)
	Current smoker	45 (16.7)	50 (14.8)
	Former smoker	39 (14.4)	48 (14.2)
	Missing data	9 (3.3)	10 (3.0)

^a^Percentages are based on the total number of patients in each analysis set

**Table 2:** Characteristics of primary disease (pancreatic carcinoma)

		FAS (*N* = 270)	SAF (*N* = 338)
Time from diagnosis of primary tumor until informed consent [days]	n (number of patients)	270	338
	Mean (SD)	168 (300)	174 (332)
	Median (Min, Max)	36 (1, 2199)	37 (1, 2383)
Time from occurrence of metastases until informed consent [days]	n (number of patients)	265	333
	Mean (SD)	71 (119)	78 (170)
	Median (Min, Max)	27 (1, 792)	29 (1, 2383)
Localization of primary tumor [n (%)] ^a,b^	Head of pancreas	157 (58.1)	190 (56.2)
	Tail of pancreas	63 (23.3)	73 (21.6)
	Body of pancreas	59 (21.9)	72 (21.3)
	Other	10 (3.7)	17 (5.0)
Localization of metastases [n (%)] ^b,c^	Liver	162 (60.7)	199 (60.5)
	Lymph nodes	71 (26.6)	83 (25.2)
	Peritoneum	46 (17.2)	56 (17.0)
	Lung	42 (15.7)	51 (15.5)
	Other	53 (19.9)	64 (19.5)
Tumor stage according to UICC [n (%)] ^a^	IV (any T, any N, M1)	219 (81.1)	274 (81.1)
	III (T4, any N, M0)	4 (1.5)	5 (1.5)
	IIb (T1-T3, N1, M0)	12 (4.4)	15 (4.4)
	IIa (T3, N0, M0)	2 (0.7)	2 (0.6)
	Ib (T2, N0, M0)	1 (0.4)	1 (0.3)
	NA	32 (11.9)	41 (12.1)
Tumor histology [n (%)] ^a^	Adenocarcinoma	249 (92.2)	306 (90.5)
	Papillary carcinoma	0	1 (0.3)
	Other	8 (3.0)	11 (3.3)
	Not performed	14 (5.2)	21 (6.2)
Biliary stent [n (%)] ^a^	No	212 (78.5)	266 (78.7)
	Yes	58 (21.5)	72 (21.3)

^a^Percentages are based on the total number of patients in each analysis set

^b^Multiple localizations of primary tumor and/or metastases were possible per patient

^c^Percentages are based on patients with data on localization of metastases available (FAS: *n* = 267; SAF: *n* = 329)

*M* distant metastasis, *N* regional lymph node, *NA* not available, *T* primary tumor, *UICC* Union for International Cancer Control

Of note, non-M1 tumor stage was documented in 7% of the patients. Most probably this reflects the stage at the time of the initial diagnosis rather than tumor stage at inclusion into the study as use of erlotinib according to the summary of product characteristics (SmPC) was mandatory in this study (see Table 2). Due to the nature of this post-approval, non-interventional study, detailed follow-up of these cases was not feasible.

### Effectiveness

**Fig. 2:**
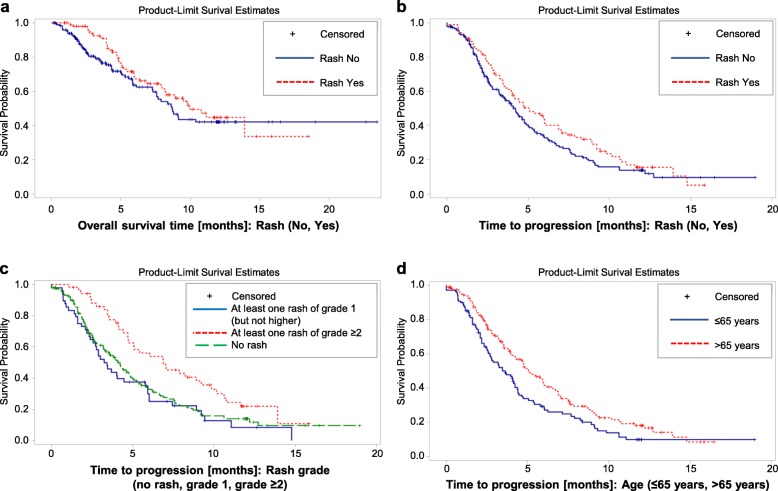
Effectiveness results – OS and PFS. **a** Overall survival stratified by rash. The dashed red curve depicts patients with any rash. The continuous blue curve shows patients without rash. **b** Progression-free survival stratified by rash. The dashed red curve depicts patients with any rash. The continuous blue curve shows patients without rash. **c** Progression-free survival stratified by the grade of cutaneous reactions. The continuous blue curve depicts patients without rash. The dashed green curve shows patients with rash grade 1. The dotted red curve displays patients with grade ≥ 2 skin reactions. **d** Progression-free survival stratified by age. The continuous blue curve shows patients aged ≤65 years. The dotted red curve shows patients aged ≥ 65 years

In the FAS population, 99 patients (36.7%) developed any skin rash, while 171 patients (63.3%) did not present with rash. Patients were stratified based on the appearance of a skin rash (dichotomous classification: rash-positive vs. rash-negative) and treatment effectiveness was calculated based on this stratification. The median overall survival (OS) of all individuals was 9.11 months with an OS of 9.93 months in rash-positive patients (*n* = 99; 36.7%) and 8.68 months in rash-negative patients (*n* = 171, 63.3%) (*p* = 0.2361, Log-Rank test) (Fig. 2a). There was no statistical difference in 1-year survival rates between the two groups (not shown). Preplanned subgroup analyses by rash grade revealed a trend towards better median OS in patients with rash grade ≥ 2 (*n* = 51, 13.91 months) when compared to patients with rash grade 1 (*n* = 48, 8.19 months) and patients without rash (*n* = 171, 8.68 months). However, this trend did not reach statistical significance (*p* = 0.0893, Log-Rank test), most likely due to the relatively small sample sizes (Additional file 1: Figure S1). Independent of skin reactions, there was a tendency of improved median OS in patients aged > 65 years (*n* = 163, 10.75 months) when compared to younger patients (*n* = 107, 8.22 months) (*p* = 0.0596, Log-Rank test) (Additional file 2: Figure S2).

In the FAS, median progression free survival (PFS) was 4.37 months. Rash-positive patients achieved a median PFS of 5.06 months, rash-negative patients a median PFS of 4.11 months (*p* = 0.1412, Log-Rank test) (Fig. 2b). Subgroup analyses revealed a statistically significant benefit in PFS of patients with rash grade ≥ 2 (*n* = 51, 6.87 months) when compared to patients with rash grade 1 (*n* = 48, 3.34 months) and patients without rash (*n* = 171, 4.11 months) (*p* = 0.0078, Log-Rank test) (Fig. 2c). Furthermore, patients aged > 65 years (*n* = 163, PFS 5.06 months) benefitted from treatment with gemcitabine/erlotinib when compared to patients ≤65 years of age (*n* = 107, PFS 3.45 months) (*p* = 0.012, Log-Rank test) (Fig. 2d).

A multivariate Cox proportional model was used to examine the influence of rash, ECOG performance status at baseline, duration of erlotinib treatment and age on OS and PFS. In the FAS, only age > 65 years proved to be associated with longer OS (hazard ratio 0.640; *p* = 0.0327) and PFS (hazard ratio 0.642; *p* = 0.0026) (Additional file 3: Table S1) and this effect was independent of skin rash. Duration of erlotinib treatment was also associated with a favorable outcome with regards to OS and PFS, most probably reflecting a negative selection of patients with early progression leading to short exposure to erlotinib. Response and DCRs did not differ significantly between patients with and without rash (not shown).

### Safety and treatment satisfaction

Out of the 338 patients in the full safety set, 39 patients (11.5%) completed the pre-specified 12-month observation period, whereas 292 patients (86.4%) terminated the study prematurely (study completion unknown for 7 patients [2.1%]). 133 patients died before regular study end, whereas 130 patients were alive at study termination with data available. Of these 130 patients, 33.1% (*n* = 43) proceeded to receive further line treatment. Twenty-nine patients were lost to follow-up (8.6%) (Table 3). The type of further line treatment was not documented.

**Table 3:** Premature study termination and deaths

Patients with a 12-month observation period (SAF) [n (%)]	39 (11.5%)
Patients with premature study termination after start of treatment, i.e. observation period < 12 months (SAF) [n (%)]	292 (86.4%)
Patients alive at study termination and with data available (SAF)	130
Main reasons for premature study termination (SAF) [n (%)]:	
Tumor progression	74 (56.9%)
Patient’s wish	21 (16.2%)
Physician’s decision	13 (10.0%)
Absent rash	9 (6.9%)
Withdrawal of informed consent	2 (1.5%)
Lost to follow-up	2 (1.5%)
Toxicity of erlotinib	1 (0.8%)
Toxicity of gemcitabine	1 (0.8%)
Other	7 (5.4%)
Patients who started a second/further-line treatment and were alive at premature termination (SAF) [n (%)]	43 (33.1%)
Deaths before regular study end (SAF)	133
Causes of death [n (%)]:	
Tumor progression	84 (63.2%)
Pancreatic cancer	29 (21.8%)
Toxicity of gemcitabine	1 (0.8%)
Unknown	11 (8.3%)
Other	8 (6.0%)
Patients who were lost to follow-up (SAF) [n (%)]	29 8.6%)

A total of 1681 adverse events (AEs) were recorded in the study. Out of the 338 patients in the SAF, 310 patients (91.7%) experienced at least one AE. A detailed listing of all the grading, outcome and type of AEs is displayed in Additional file 4: Table S2 and Additional file 5: Table S3. Most AEs were CTC grade 1 to 3. In total, 222 patients (65.7%) experienced AEs related to erlotinib, while 156 patients (46.2%) had AEs related to gemcitabine. A special focus was laid on skin-related AEs. In the SAF, 176 patients (52.1%) experienced skin-related side effects, all of which were CTC grade 1 to 3 (Additional file 6: Table S4). Overall, serious adverse events (SAEs) were documented in approximately half of the SAF (50.6%, *n* = 171) including patients, for whom a tumor progression was documented as SAE (19.2%, *n* = 65). 68 patients (20.1%) had a fatal SAE including 22 patients (6.5%) for whom progression of the underlying tumor disease was documented as related to the fatal SAE. According to the definition of SAEs in the protocol, a fatal or life-threatening event had to be documented as an SAE.

**Fig. 3:**
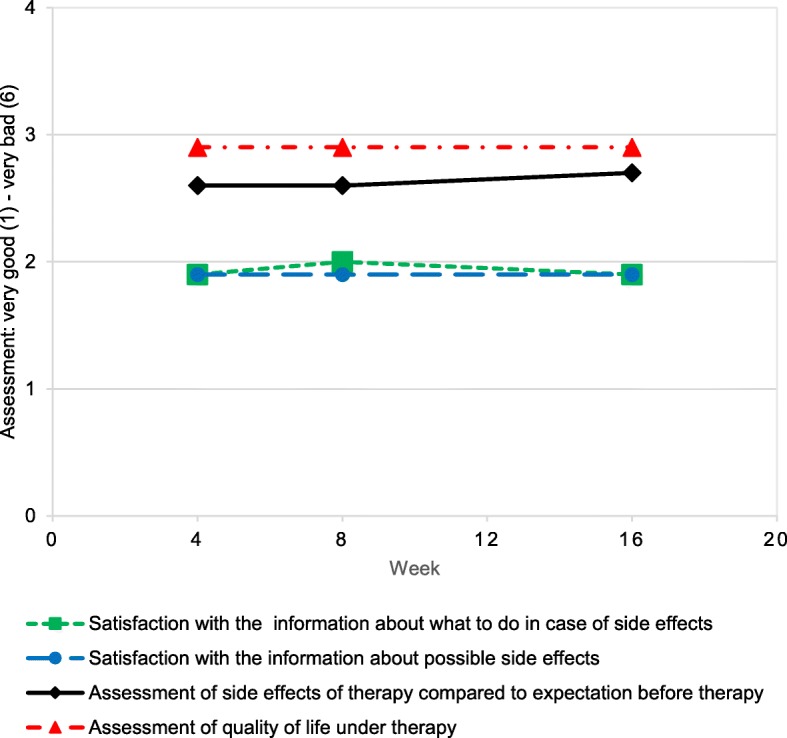
Treatment satisfaction over time. The dashed blue line (circles) depicts patient satisfaction with the information regarding possible side effects. The dashed green line (squares) shows patient satisfaction with the information about what to do in case of side effects. The continuous black line (diamonds) shows patients’ assessment of experienced side effects in relation to side effects expected before therapy. The dashed red line (triangles) depicts patients’ assessment of quality of life under therapy. Data are depicted as mean. The scale ranged from 1 (very good) to 6 (very bad)

During treatment, patients self-reported their treatment satisfaction and assessment of side effects at weeks 4, 8 and 16 on a numerical scale from one (very satisfied/less than expected/very good) to six (not satisfied/more than expected/very poor). At week 4, patients were satisfied regarding treatment side effects (mean score: 1.9 ± 0.9, *n* = 180) and with the information about what to do in case of occurring side effects (mean score: 1.9 ± 0.8, *n* = 179). With regard to the severity of side effects, patients reported the side effects slightly less severe than what they had expected before the therapy (mean score: 2.6 ± 1.1, *n* = 179). Patients rated their quality of life as fair to good (mean score: 2.9 ± 1.1, *n* = 180). Overall, these parameters remained virtually unchanged during the course of the study with exception of the patients’ assessment of the experienced side effects in comparison to anticipated side effects; here patients reported an incremental increase in intensity (Fig. 3).

## Discussion

We report the results of a multi-institutional phase IV study of gemcitabine/erlotinib in the treatment of metastatic pancreatic cancer. Based on previous reports [7–11], the overarching goal of this study was to demonstrate the clinical effectiveness of gemcitabine/erlotinib stratified by the onset of cutaneous reactions to erlotinib. Applying a dichotomous classification (rash-positive vs. rash-negative), the study did not meet its primary endpoint, as there was no difference in OS or PFS in patients with rash compared to patients without skin reactions. With regards to the severity of cutaneous reactions, previous studies described a grade-dependent benefit from erlotinib with the greatest benefit in patients with rash grade ≥ 2 [7–10]. In line with these results, patients with rash grade ≥ 2 showed a trend towards better OS compared to patients with no rash or rash grade 1. However, due to the relatively small sample size, this trend did not reach statistical significance. The predictive value of the severity of rash was further underscored by the fact, that patients with grade ≥ 2 rash experienced a statistically significant increase in PFS.

Irrespective of skin reactions, patients aged 65 years or older had an improved PFS under treatment with gemcitabine/erlotinib when compared to younger patients. Likewise, there was a trend towards better OS in older patients, which however did not reach statistical significance (*p* = 0.0596, Log-Rank test). As older patients tend to have inferior clinical outcomes in metastatic pancreatic cancer [15], this subgroup might especially benefit from the combination of gemcitabine/erlotinib. Considering the favorable safety profile of gemcitabine/erlotinib and the potential of cutaneous reactions to predict treatment outcome, treatment with the combination tested here might offer a therapeutical alternative in the management of elderly patients with metastatic pancreatic cancer deemed unfit for other, more toxic, combinational regimens.

In order to adequately place the data presented here into the context of previous publications, differences in study design and the patient population analyzed need to be considered. The interventional clinical trials mentioned above [7–10] were conducted in a first line setting and included close to 25% of patients with only locally advanced disease. The cohort presented here exclusively included patients with metastatic disease and more than a fifth (21.5%) of patients had received previous palliative chemotherapy in the metastatic setting. These profound differences might explain why this non-interventional study did not meet its primary endpoint with regards to overall survival stratified by rash.

The data on AEs and patient satisfaction reported here are well in line with previous reports [2, 7, 10]; the study did not yield any new safety signals. At present, second [16, 17] and third line regimens for the treatment of metastatic pancreatic cancer are more frequently administered and clinicians think in terms of sequences. Accordingly, it is important to consider cumulative toxicity during each line of treatment to ensure optimal results over multiple lines of therapy [14]. Thus, a general disregard of treatment modalities such as gemcitabine/erlotinib with a favorable safety profile and potential benefit, even if this benefit may be restricted to specific subgroups, does not seem to be justified at this point.

## Conclusions

In this large, multi-center study, treatment of patients with metastatic pancreatic cancer with gemcitabine/erlotinib was safe and well tolerated. Based on the stratification used (rash-positive versus rash-negative), treatment with gemcitabine/erlotinib did not prolong OS and PFS in the FAS. However, older patients and patients with rash grade ≥ 2 experienced a significantly favorable PFS. Moreover, older patients and patients with rash grade ≥ 2 showed a trend towards better median OS that, however, was not significant. As further line treatment of metastatic pancreatic cancer becomes more frequent, gemcitabine/erlotinib treatment might remain an option in selected subgroups suffering from this devastating disease.

## Supplementary information

**Additional file 1: Figure S1**. Overall survival stratified by the grade of cutaneous reactions. The dashed green curve depicts patients without rash. The continuous blue curve shows patients with rash grade 1. The dotted red curve displays patients with grade ≥ 2 skin reactions.

**Additional file 2: Figure S2.** Overall survival stratified by age. The continuous blue curve shows patients aged ≤65 years. The dotted red curve shows patients aged > 65 years.

**Additional file 3: Table S1.** Results of multivariate Cox proportional hazard models for overall and progression-free survival.

**Additional file 4: Table S2.** Summary and grade of adverse events in the study population.

**Additional file 5: Table S3.** Type and frequency of adverse events and adverse drug reactions in the study population.

**Additional file 6: Table S4.** Type, frequency and grade of skin-related adverse events in the study population.

## Data Availability

Qualified researchers may request access to individual patient level data through the clinical study data request platform (www.clinicalstudydatarequest.com). Further details on Roche’s criteria for eligible studies are available here: https://clinicalstudydatarequest.com/Study-Sponsors/Study-Sponsors-Roche.aspx For further detail on Roche’s Global Policy on the Sharing of Clinical Information and how to request access to related clinical study documents, see here: https://www.roche.com/research_and_development/who_we_are_how_we_work/clinical_trials/our_commitment_to_data_sharing.htm

## Abbreviations

AE: Adverse event
AMG: Arzneimittelgesetz (German Drug Law)
CTCAE: Common Terminology Criteria for Adverse Events
DCR: Disease control rate
ECOG: Eastern Cooperative Oncology Group
FAS: Full analysis set
OS: Overall survival
PDAC: Pancreatic ductal adenocarcinoma
PFS: Progression-free survival
SAE: Serious adverse event
SAF: Safety analysis set
SmPC: Summary of product characteristics
