# Supplementary figures and images for: Correlation of skin rash and overall survival in patients with pancreatic cancer treated with gemcitabine and erlotinib – results from a non-interventional multi-center study
Source: BMC Cancer. 2020 Feb 24;20:155. doi: 10.1186/s12885-020-6636-7 (PMC7041266; doi:10.1186/s12885-020-6636-7)

Product-Limit Survival Estimates

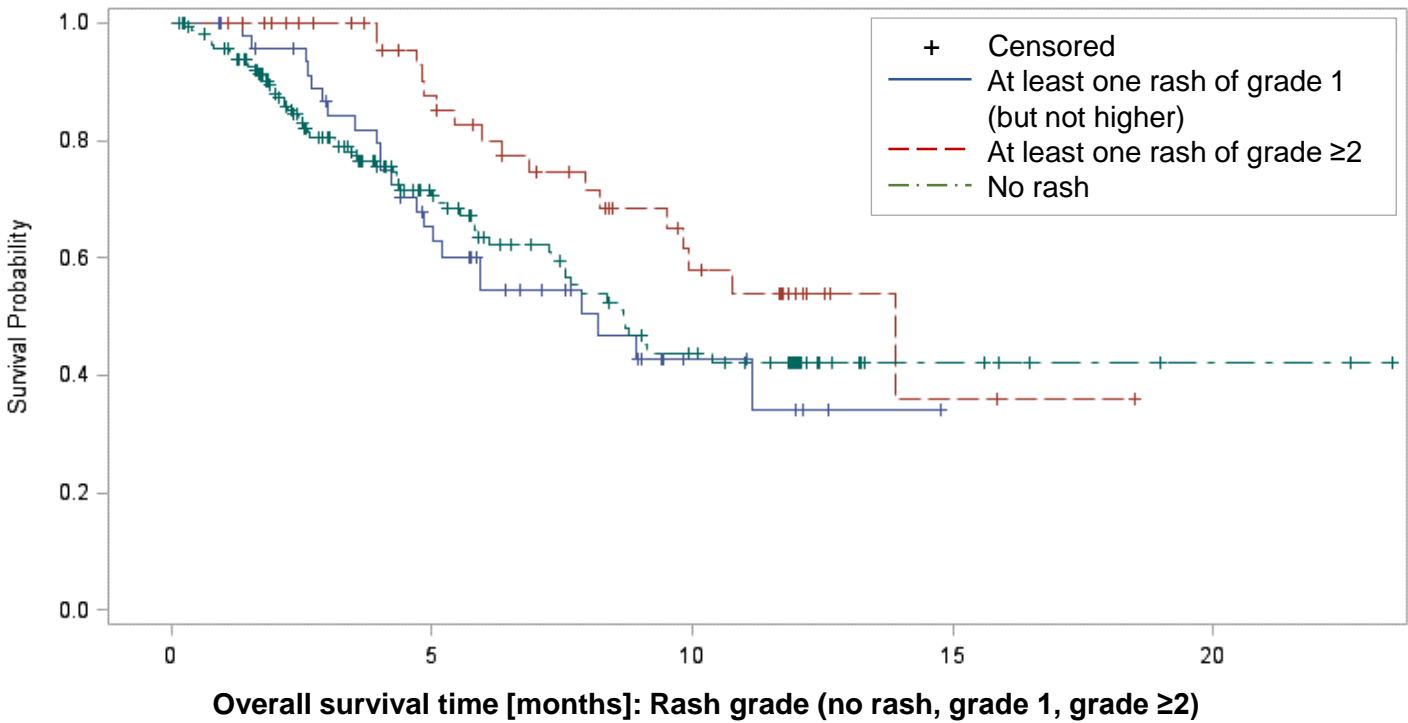

Supplement: Supplementary file 1 — Additional file 1: Figure S1. Overall survival stratified by the grade of cutaneous reactions. The dashed green curve depicts patients without rash. The continuous blue curve shows patients with rash grade 1. The dotted red curve displays patients with grade ≥ 2 skin reactions. [file 12885_2020_6636_MOESM1_ESM.pdf]

Product-Limit Survival Estimates

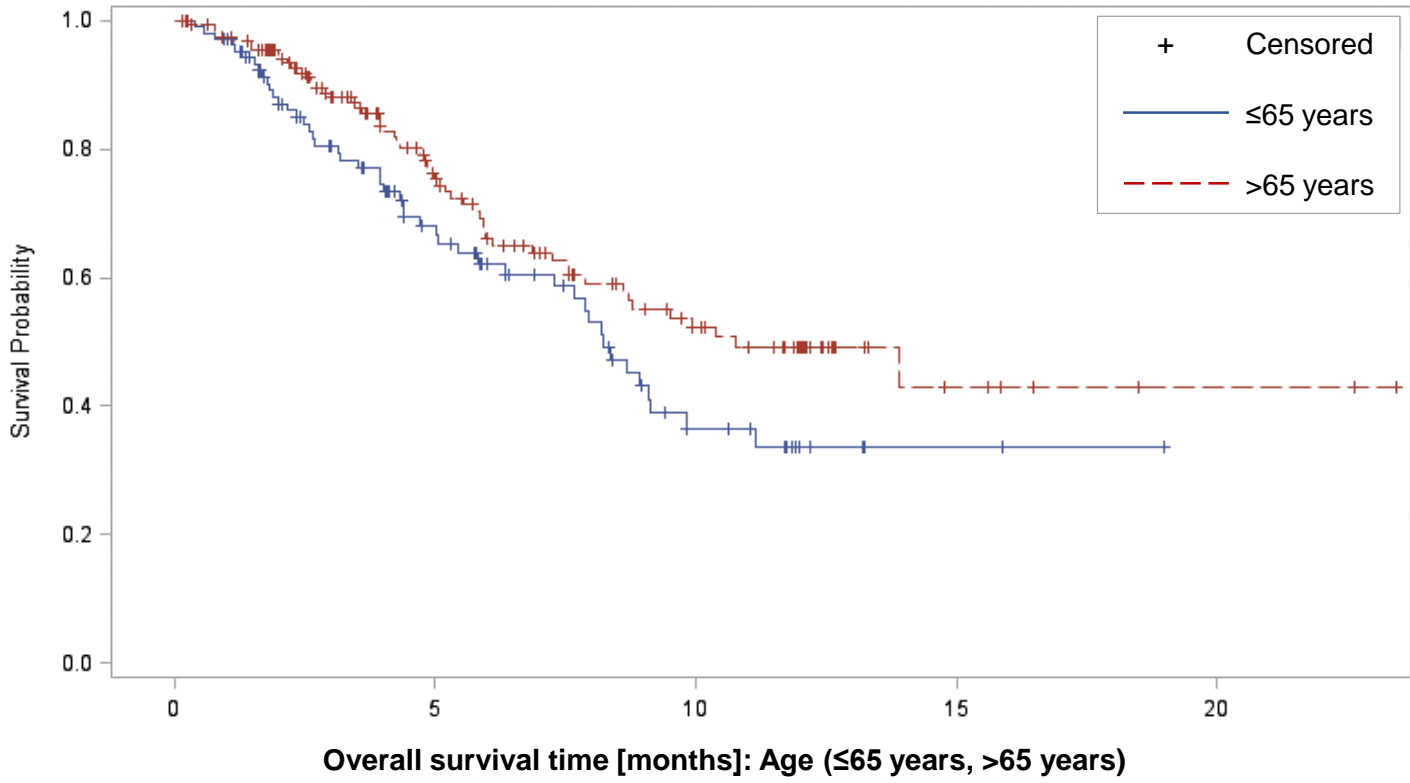

Supplement: Supplementary file 2 — Additional file 2: Figure S2. Overall survival stratified by age. The continuous blue curve shows patients aged ≤65 years. The dotted red curve shows patients aged > 65 years. [file 12885_2020_6636_MOESM2_ESM.pdf]
