# Supplementary material for: Correlation of skin rash and overall survival in patients with pancreatic cancer treated with gemcitabine and erlotinib – results from a non-interventional multi-center study
Source: BMC Cancer. 2020 Feb 24;20:155. doi: 10.1186/s12885-020-6636-7 (PMC7041266; doi:10.1186/s12885-020-6636-7)
Supplement: Supplementary file 3 — Additional file 3: Table S1. Results of multivariate Cox proportional hazard models for overall and progression-free survival. [file 12885_2020_6636_MOESM3_ESM.docx]

|  |  | **FAS (N=270)** | |
| --- | --- | --- | --- |
| **Outcome variable: overall survival time** | | Hazard ratio | p value |
| Independent variables: | |  |  |
| Rash: no vs. yes | | 0.978 | 0.9182 |
| Duration of erlotinib treatment: ≤ 8 weeks vs. > 8 weeks | | 0.202 | <0.0001 |
| ECOG performance status at baseline: 0-1 vs. 2 | | 0.895 | 0.7397 |
| Age: ≤ 65 years vs. > 65 years | | 0.640 | 0.0327 |
| **Outcome variable: time to progression** | |  |  |
| Independent variables: | |  |  |
| Rash: no vs. yes | | 0.926 | 0.6156 |
| Duration of erlotinib treatment: ≤ 8 weeks vs. > 8 weeks | | 0.393 | <0.0001 |
| ECOG performance status at baseline: 0-1 vs. 2 | | 1.221 | 0.4188 |
| Age: ≤ 65 years vs. > 65 years | | 0.642 | 0.0026 |
