# Supplementary material for: Correlation of skin rash and overall survival in patients with pancreatic cancer treated with gemcitabine and erlotinib – results from a non-interventional multi-center study
Source: BMC Cancer. 2020 Feb 24;20:155. doi: 10.1186/s12885-020-6636-7 (PMC7041266; doi:10.1186/s12885-020-6636-7)
Supplement: Supplementary file 4 — Additional file 4: Table S2. Summary and grade of adverse events in the study population. [file 12885_2020_6636_MOESM4_ESM.docx]

|  | **SAF (N=338)** |
| --- | --- |
| Total number of adverse events | 1681 |
| Patients with at least 1 adverse event [n (%)]^a^ | 310 (91.7) |
| Patients with adverse events by intensity [n (%)]^a,b^ |  |
| CTC grade 1 | 213 (63.0) |
| CTC grade 2 | 208 (61.5) |
| CTC grade 3 | 130 (38.5) |
| CTC grade 4 | 5 ( 1.5) |
| CTC grade 5 | 68 (20.1) |
| Missing data | 109 (32.2) |
| Patients with adverse events related to gemcitabine [% (n)]^a^ | 156 (46.2) |
| Patients with adverse events related to erlotinib [n (%)]^a^ | 222 (65.7) |
| Patients with adverse events by outcome [n (%)]^a,b^ |  |
| Recovered | 253 (74.9) |
| Remaining harm | 4 ( 1.2) |
| Not yet recovered | 132 (39.1) |
| Exitus^c^ | 68 (20.1) |
| Unknown / Missing data | 147 (43.5) |
| Patients with at least 1 serious adverse event [n (%)]^a^ | 171 (50.6) |
| Patients with adverse event ‘rash’ [% (n)]^a^ | 174 (51.5) |
| Patients with adverse events requiring therapy [n (%)]^a,b^ | 245 (72.5) |

^a^ Percentages are based on the total number of patients in the SAF

^b^ Multiple mentions per patient possible

^c^ Overall, 133 patients died in the present study. However, as death due to a progression of the underlying tumor disease was defined as an effectiveness endpoint in this study setting, no additional documentation as an adverse event was required.
