# Supplementary material for: Correlation of skin rash and overall survival in patients with pancreatic cancer treated with gemcitabine and erlotinib – results from a non-interventional multi-center study
Source: BMC Cancer. 2020 Feb 24;20:155. doi: 10.1186/s12885-020-6636-7 (PMC7041266; doi:10.1186/s12885-020-6636-7)
Supplement: Supplementary file 5 — Additional file 5: Table S3. Type and frequency of adverse events and adverse drug reactions in the study population. [file 12885_2020_6636_MOESM5_ESM.docx]

| **MedDRA System Organ Class** | **SAF (N=338)** | |
| --- | --- | --- |
|  | **AEs [n (%)]^a^** | **ADRs [n (%)]^a^** |
| Skin and subcutaneous tissue disorders | 190 (56.2) | 176 (52.1) |
| General disorders and administration site conditions | 166 (49.1) | 43 (12.7) |
| Gastrointestinal disorders | 152 (45.0) | 75 (22.2) |
| Investigations | 109 (32.2) | 24 ( 7.1) |
| Neoplasms benign, malignant and unspecified (incl. cysts and polyps) | 65 (19.2) | 1 ( 0.3) |
| Infections and infestations | 62 (18.3) | 5 ( 1.5) |
| Respiratory, thoracic and mediastinal disorders | 43 (12.7) | 5 ( 1.5) |
| Metabolism and nutrition disorders | 38 (11.2) | 14 ( 4.1) |
| Nervous system disorders | 35 (10.4) | 12 ( 3.6) |
| Vascular disorders | 30 ( 8.9) | 9 ( 2.7) |
| Hepatobiliary disorders | 26 ( 7.7) | 0 ( 0.0) |
| Psychiatric disorders | 17 ( 5.0) | 3 ( 0.9) |
| Blood and lymphatic system disorders | 16 ( 4.7) | 3 ( 0.9) |
| Eye disorders | 10 ( 3.0) | 4 ( 1.2) |
| Cardiac disorders | 8 ( 2.4) | 2 ( 0.6) |
| Renal und urinary disorders | 8 ( 2.4) | 0 ( 0.0) |
| Injury, poisoning and procedural complications | 6 ( 1.8) | 2 ( 0.6) |
| Musculoskeletal and connective tissue disorders | 4 ( 1.2) | 0 ( 0.0) |
| Congenital, familial and genetic disorders | 1 ( 0.3) | 0 ( 0.0) |
| Reproductive system and breast disorders | 1 ( 0.3) | 0 ( 0.0) |
| Surgical and medical procedures | 1 ( 0.3) | 0 ( 0.0) |

^a^ Percentages are based on the total number of patients (SAF).

ADR = Adverse drug reaction (AE with causal relationship to erlotinib); AE = Adverse event; MedDRA = Medical Dictionary for Regulatory Activities; SAF = Safety analysis set.
