# Supplementary material for: Correlation of skin rash and overall survival in patients with pancreatic cancer treated with gemcitabine and erlotinib – results from a non-interventional multi-center study
Source: BMC Cancer. 2020 Feb 24;20:155. doi: 10.1186/s12885-020-6636-7 (PMC7041266; doi:10.1186/s12885-020-6636-7)
Supplement: Supplementary file 6 — Additional file 6: Table S4. Type, frequency and grade of skin-related adverse events in the study population. [file 12885_2020_6636_MOESM6_ESM.docx]

| **MedDRA Preferred Term [n (%)]^a^** | **SAF (N=338)** | | | | |
| --- | --- | --- | --- | --- | --- |
|  | **Grade** | | | | |
|  | **1** | **2** | **3** | **4** | **5** |
| Papulopustular eczema | 78 (23.1) | 62 (18.3) | 6 (1.8) | 0 (0.0) | 0 (0.0) |
| Dry skin | 59 (17.5) | 26 ( 7.7) | 0 (0.0) | 0 (0.0) | 0 (0.0) |
| Paronychia | 9 ( 2.7) | 5 ( 1.5) | 1 (0.3) | 0 (0.0) | 0 (0.0) |

^a^ Percentages are based on the total number of patients (SAF).

Classification modified according to NCI-CTCAE Version 4.03.
MedDRA = Medical Dictionary for Regulatory Activities; NCI-CTCAE = National Cancer Institute - Common Terminology Criteria for Adverse Events; SAF = Safety analysis set.
